# Effects of Strength Training on Cognitive Function, Brain-Derived Neurotrophic Factor and Insulin-Like Growth Factor 1 in Highly-Trained Young Female Soccer Players

**DOI:** 10.1186/s40798-026-01103-z

**Published:** 2026-09-18

**Authors:** Mariem Bousselmi, Hassane Zouhal, Manel Darragi, Houssem M. Karamti, Ahlem Ben Hmid, Imen Zamali, Mélika Ben Ahmed, Asma Krir, Sghaeir Zouita, Ismail Laher, Anthony C. Hackney, Urs Granacher, Amira Ben Moussa Zouita

**Affiliations:** 1 Higher Institute of Sport and Physical Education of SFAX, University of Sfax, 3O27 Sfax, Tunisia; 2 Research Laboratory (LR23JS01) “Sport Performance, Health & Society”, Higher Institute of Sport and Physical Education of Ksar Said, 1000 Tunis, Tunisia; 3 International Institute of Sport Sciences (2I2S), 35000 Rennes, France; 4 Clinical Immunology Department, Pasteur Institute of Tunis, 1000 Tunis, Tunisia; 5 Laboratory of Transmission, Control and Immunobiology of Infections (LR11IPT02), Pasteur Institute of Tunis, 1000 Tunis, Tunisia; 6 Faculty of Medicine of Tunis, Tunis El Manar University, 1000 Tunis, Tunisia; 7 Department of Clinical Biochemistry, Pasteur Institute of Tunis, 1000 Tunis, Tunisia; 8 Higher Institute of Sport and Physical Education of Ksar Said, University of Manouba, 1000 Tunis, Tunisia; 9 Department of Anesthesiology, Pharmacology and Therapeutics, The University of British Columbia, Vancouver, Canada; 10 Department of Exercise and Sport Science, University of North Carolina, Chapel Hill, North Carolina USA; 11 Department of Sport and Sport Science, Exercise and Human Movement Science, University of Freiburg, Freiburg, Germany

**Keywords:** Weight-bearing exercise, Resistance training, BDNF, IGF-1, Youth, Stroop, Football

## Abstract

**Background:**

Female soccer continues to gain global popularity. Selected components of physical fitness (e.g., muscle power, change-of-direction [CoD] speed) and cognitive function (e.g., attentional demand, training-induced) play an important role in soccer performance and should, therefore, be systematically developed.

**Objectives:**

In this study, we examined the effects of 12 weeks of strength training (ST) on selected measures of physical fitness, soccer performance, cognitive function, and selected markers of neuroplasticity in young female soccer players.

**Methods:**

Twenty-two highly-trained female soccer players with Tier 3 training and performance caliber aged 14.9±0.8 years were randomly assigned to a strength training group (SG: *n* = 11) or an active control group (CG: *n* = 11). During the training period, SG performed two weekly upper and lower body ST sessions (3 sets, 12 repetitions per set) at an intensity of 60–80% of the one-repetition-maximum (1-RM) and three soccer-specific training sessions (tactical–technical drills). Players from CG continued their regular soccer-specific training routine consisting of five weekly sessions. Training volumes were similar between groups. Pre- and post-training tests were conducted for the assessment of body composition (e.g., body mass, skinfold site measurements), muscle strength (1-RM bench press/Lat pull-down/leg press),the Loughborough Soccer Passing Test (LSPT), cognitive function (Stroop test), and selected markers of blood neurotrophins, such as basal brain-derived neurotrophic factor (BDNF) and insulin-like growth factor 1 (IGF-1) serum concentrations.

**Results:**

The following findings from post hoc analyses are based on significant (*p* <.05) group-by-time interactions. Post hoc analyses revealed ST-related increases in muscle strength for 1-RM bench press (*p* <0.001; η_p_^2^=0.41), lat pull-down (*p* <0.001; η_p_^2^=0.43), and leg press (*p* <0.001; η_p_^2^=0.36) and improvements in soccer-specific performance for the LSPT in the SG (*p* =0.007; η_p_^2^=0.22). Post hoc tests indicated improved cognitive function (Stroop test) in the SG (*p* <0.001; η_p_^2^=0.56) but not in the CG. There were no significant group-by-time interactions for neuroplasticity markers, such as BDNF and IGF-1 basal serum concentrations (*p*>0.05; η_p_^2^=0.00–0.04).

**Conclusions:**

A 12-week in-season ST program with two weekly sessions improved measures of physical fitness, soccer-specific performance, and cognitive function but not biomarkers of neuroplasticity in young highly-trained female soccer players. Accordingly, we recommend ST should be implemented to improve physical fitness and cognitive function of young female soccer athletes.

**Supplementary Information:**

The online version contains supplementary material available at https://doi.org/10.1186/s40798-026-01103-z.

## Key Points

- Two weekly strength training (ST) sessions over an in-season training period of 12 weeks improved physical fitness (1-RM bench press/lat pull-down/leg press) and soccer-specific performance (LSPT) in young and highly-trained female soccer players.
- ST improved cognitive function (STROOP) in young and highly-trained female soccer players.
- ST did not result in any significant differences in serum concentrations of neuroplasticity markers (BDNF, IGF-1) in young and highly-trained female soccer players.

**Supplementary Information:**

The online version contains supplementary material available at https://doi.org/10.1186/s40798-026-01103-z.

## Background

Soccer continues to surge in global popularity, resulting in large increases in the overall percentage of female (professional) soccer players [1]. The intermittent character of soccer matches affords multi-factorial physical fitness demands (e.g., linear sprint and change-of-direction [COD] speed), technical (e.g., ball dribbling) and tactical skills (e.g., pressing), and cognitive function (e.g., decision-making) to successfully perform during match play [2, 3].

Authors of previous studies have examined the effects of different training modalities (e.g., strength [ST], endurance training) on measures of physical fitness and sport-specific performance in female soccer players. For instance, Pacholek and Zemková [4] investigated the impact of ST on muscle strength and power in elite female soccer players (Tier 3 training and performance caliber) [5] aged 20.2 ± 3.3 years. These authors reported a training-induced increase in proxies of muscle strength 1-RM, half squat, bench pull) and muscle power (e.g., vertical jump height).

To successfully perform during soccer matches, players need adequate levels of physical fitness, technical, tactical skills and high levels of cognitive function to be able to make quick decisions during complex soccer match situations. During soccer training and matches, players should be alert for different external stimuli, such as opponent and team-mate directions and positions, and ball passing situations [3, 6]. For instance, athletes should be able to concurrently dribble or pass the ball at proficient skill level while observing the pitch [3].

Accordingly, soccer players are constantly acting in so-called multi-task situations under time pressure with the primary task being a motor or soccer-specific task and the secondary a cognitive task [3]. Performance in these multi-task situations affords divided attention and quick training-induced. A previous study has shown that executive function as tested by the Stroop test has the potential to differ between soccer players of high versus low expertise level [6]. In addition, there is evidence that the ability to perform multi-tasks concurrently can be improved through adequate training modalities in the general population (Tier 0 training and performance caliber) [8] and in soccer players (Tier 2–3 training and performance caliber) [6, 7].

Blood neurotrophins such as BDNF and proteins with neurotrophic properties such as IGF-1 are considered to be of major importance for mediating the benefits of exercise training on the brain by the induction of central and peripheral growth factor cascades [9–11]. Several studies using animal models demonstrated that exercise training results in neurocyte neogenesis, long-term potentiation and long-term plasticity, which are regulated by BDNF and IGF-1 [12–14]. Other studies involving humans reported increases of IGF-1 serum concentrations after ST [15, 16], whereas responses of basal IGF-1 concentrations to endurance training were contradictory depending on the exercise intensity [17, 18]. However, there appear to be no studies that have examined the effects of ST on cognitive function in young female soccer players. Moreover, the underlying physiological mechanisms for ST-induced improvements in cognitive function, such as BDNF or IGF-1 serum concentrations, are currently not well-understood. Findings from a meta-analysis including 50 studies [19] indicated that ST performed at high intensities has the potential to improve markers of neuroplasticity (e.g., BDNF), particularly in young and healthy individuals. In another meta-analysis, Kidgell et al. [20] observed similar findings as Hortobagyi et al. [19] and speculated that cellular and molecular pathways such as BDNF and IGF-1 could be responsible for ST-related improvements in cognitive function [21].

Here, we aimed to examine the effects of 12 weeks of ST on physical fitness, soccer-specific skills, and cognitive function, and selected markers of blood neurotrophins (BDNF, IGF-1 concentrations) in young and highly-trained female soccer players. Based on the available literature [19–23], we hypothesized that ST improves physical fitness [4, 24, 25], executive function [6, 7], and the underlying markers of neuroplasticity [19–22]. It should be noted that this study is part of a global research project focusing on the same study population and using a similar experimental protocol. This research project consists of several studies with different objectives. The study by Bousselmi et al. [26] examined the effects of 12 weeks of ST on maximal dynamic strength, muscle damage biomarkers (LDH, CPK), and inflammatory biomarkers (TNF-α, IL-6) in young elite female soccer players. Darragi et al. [27] studied the effect of this training program on physical parameters and injury incidence in the same population. Another study is also examines the effect of this same ST program on hormonal fluctuations in the same population. Finally, the current study reported herein examines the effect of the same ST program on cognitive function, BDNF and IGF-1 in this population.

## Methods

### Participants

Although this study, as well as those by Darragi et al. [27] and Bousselmi et al. [26] are part of the same overall research project, the number of samples (i.e., subjects) differs from one study to another due to the availability of data and blood tests. For the credibility of the study, an additional document has been provided with details.

A statistical power tool (G.power, version 3.1, University of Düsseldorf, Germany) was used to estimate the sample size needed to achieve significant group-by-time interactions for the outcome parameter Stroop test. As input parameters, we included an alpha level of 0.01 and 90% statistical power. The effect size Cohen’s *f* = 0.54 was taken from a related study with similar study design and outcome [28]. The power analysis indicated that a total of 18 players would be needed to achieve significant group-by-group interactions. Accordingly, 22 players were recruited to account for potential dropouts due to injuries or illness.

Female soccer players aged 14.9±0.8 years from the Tunisian national U15 soccer team were recruited to participate in this study. According to the definition of training and performance caliber by McKay [5], the enrolled athletes can be classified as Tier 3 training and performance caliber (highly-trained). Players were randomly allocated using a simple randomization process to either a SG (*n* = 11) or an active CG, conducting soccer-specific training only (*n* = 11). Players’ allocation to the experimental groups was performed using a computer-generated random sequence by an investigator not involved in testing and training aspects of the study.

### Study Procedures

The players performed five weekly training sessions, each lasting 90 min, and played one competitive game per week. The inclusion criteria comprised a minimum of 2 years of systematic soccer training at the national level. The exclusion criteria comprised musculoskeletal injuries occurring before or during the intervention, attendance at fewer than 85% of the scheduled training sessions, and previous participation in systematic ST. All players participated in the soccer academy throughout the season, except on weekends when they returned to their families or joined the soccer (national) team to compete in matches. The soccer academy’s nutritional staff members monitored dietary and hydration practices, providing athletes with nutritional guidelines for weekend meals. The involved coaches were blinded to the aim of the study.

Pubertal development, as marked by the appearance of secondary sexual characteristics, was determined by a medical doctor experienced in the assessment of these characteristics according to the Tanner method [29] and was used to detect physiological advancement and evaluate changes using the Tanner stages. According to their pubescent status, the soccer players involved in the current study belonged to Tanner stage 3 and can be considered pubertal [29].

The study was conducted in accordance with the latest version of the Declaration of Helsinki and it was approved by the Ethics Committee of the University of Sfax, Tunisia (CPP SUD N°0493/2023) and the National Ethics Committee of the Tunisian Soccer Federation. The study was retrospectively registered in the clinical trials registry on March 21st 2025 (Pan African clinical trials Registry) under the identification number “PACTR202504552619186” accessible via the following link: https://pactr.samrc.ac.za. This study adheres to the CONSORT guidelines.

Study participants and their legal representatives were informed about the testing protocols but blinded to the aims of the experiment before signing written informed consent. Players could withdraw study participation at any time without providing reasons and experiencing any consequences.

**Fig. 1:**
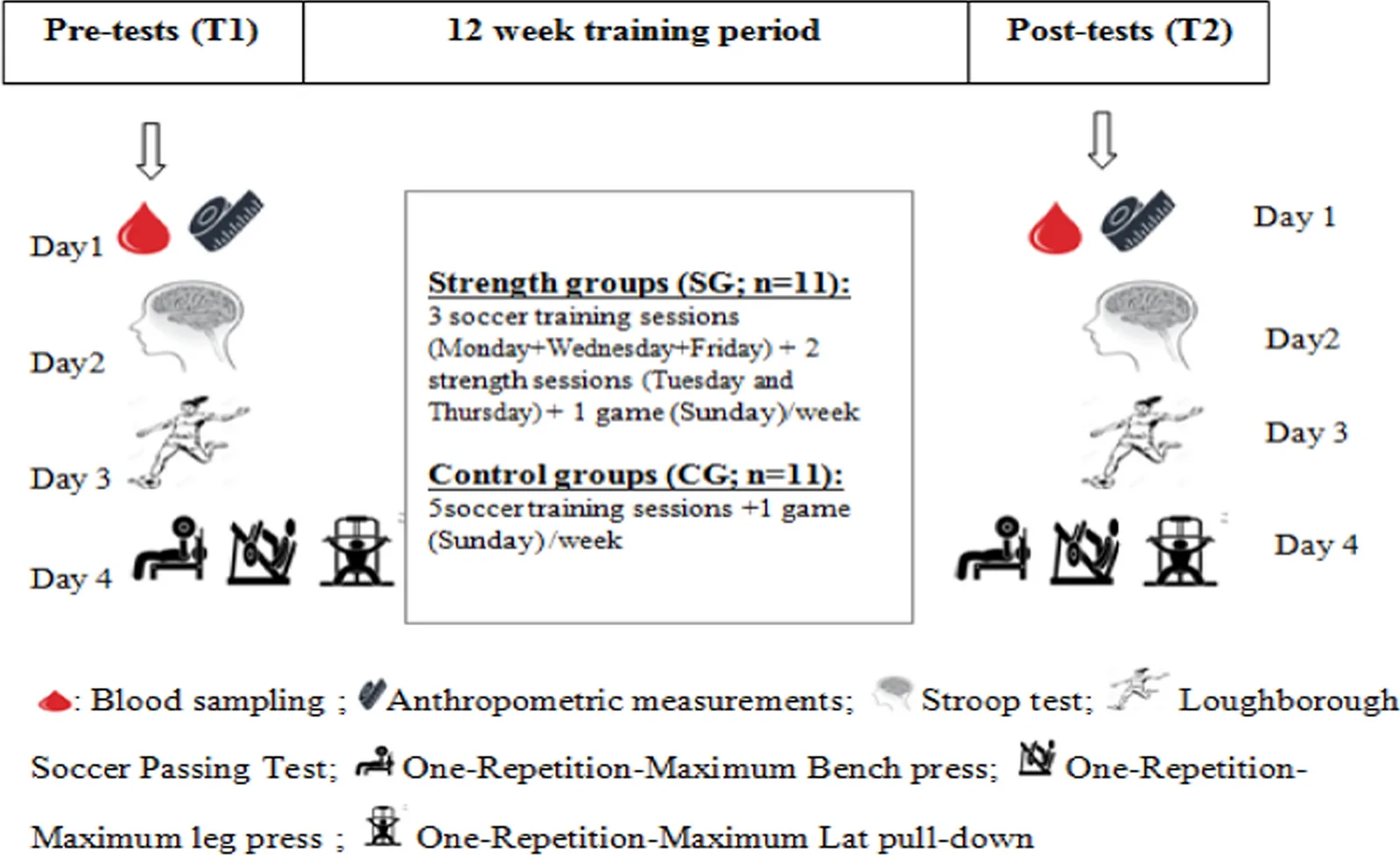
Study design of the randomized controlled trial

Pre (T1), post (T2) the 12-week intervention period, tests were conducted for the assessment of physical fitness, cognitive function, and markers of neuroplasticity (Fig. 1). All tests were performed at the same time of day, in the same order, with the same assessor, who was blinded to the aims of the study. All players were familiar with the tests.

### Anthropometrics and Body Composition

Body mass was determined using a digital scale (Beurrer BF 180, GmBH, Ulm, Germany) with an accuracy of 0.1 kg, and body height was measured with a SECA stadiometer with an accuracy of 0.1 cm. A skinfold forceps (Harpenden type, Switzerland) was used to calculate relative fat mass (%) according to the equation of Durnin and Womersley [30]. The body mass index (BMI) was calculated using body mass divided by body height squared. All measurements were taken in the morning in the fasted state. The characteristics of the study sample are illustrated in Table 1.

**Table 1:** Characteristics of the Study Sample

Variables	SG (*n* = 11)	CG (*n* = 11)
Age (years)	14.8 ± 0.7	15.1 ± 0.9
Body height (cm)	164.5 ± 6.5	164.0 ± 0.6
Body mass (kg)	55.8 ± 8.2	56.8 ± 7.7
BMI (kg/m^2^)	20.5 ± 2.8	21.1 ± 2.3
Body fat (%)	25.5 ± 2.4	26.4 ± 2.4

*SG* strength training group, *CG* active control group, *BMI* body mass index

### Physical Fitness Tests

#### Muscle Strength

Maximal dynamic strength was assessed using 1-RM tests on the leg press, bench press, and lat pull-down machines (Matrix, Houdan, France). The 1-RM was determined following the recommendations proposed by the American College of Sports Medicine (ACSM) [31]. Following a general dynamic warm-up, 1-RM testing began with three progressive lifts with incrementally increasing loads at 40, 75, and 85% of the estimated 1-RM. The number of repetitions diminished as the session advanced (10→6→3). To determine the 1 RM, the initial effort was conducted with a load of 5% under the estimated 1-RM. Upon a successful lift, the load was augmented by roughly 5%, and the test concluded when the participant was unable to lift the load after 2–3 attempts. The heaviest load that was successfully lifted once was recorded as the 1-RM. Participants recovered for 3 min between lifts [32].

#### Loughborough Soccer Passing Test

**Fig. 2:**
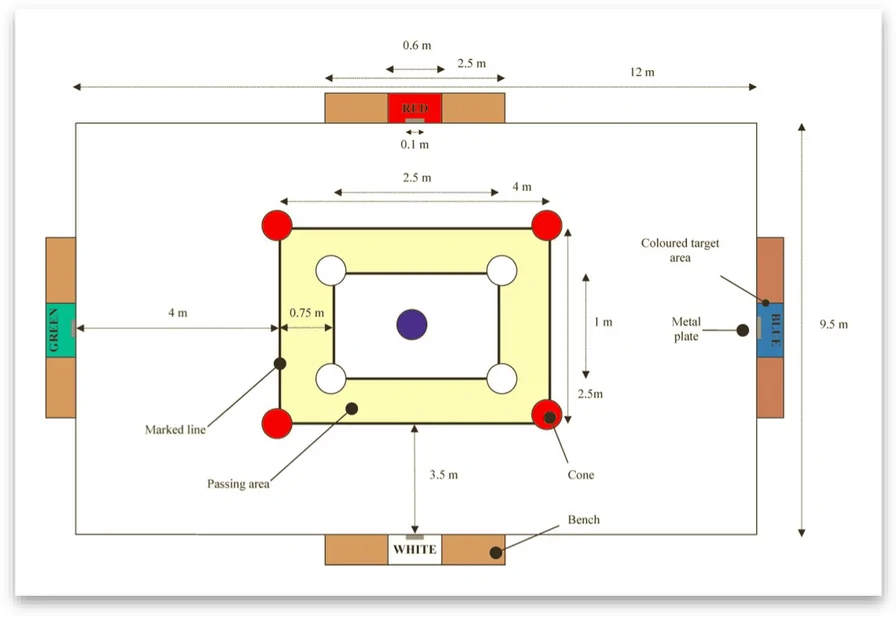
Schematic illustration of the Loughborough Soccer Passing Test (LSPT) [33]

The Loughborough Soccer Passing Test was developed and validated for research purposes by Ali et al. [33] to assess soccer-specific technical performance. The players started the LSPT with the ball at the center point of the test space, and then they were asked to complete 16 passes (about 4 m distance) against benches placed in a rectangle around the player (Fig. 2). A colored piece of cardboard (60×30 cm) was attached to the center of each bench, serving as a target area (red, blue, green, and white), and passes were made in one of the four random color orders selected by an interviewer. Players were asked to complete all 16 passes as quickly as possible while attempting to minimizing errors. The sequence of passes was determined by one of the eight trial commands randomly generated by the investigators, such that each trial consisted of eight long passes (4 m; green and blue) and eight short passes (3.5 m; white and red). LSPT performance includes the original time to complete all 16 passes, penalty time (time added for errors, inaccurate passes), and performance time (original time + penalty time). The penalty time was calculated using the following criteria: +5 s for completely missing the bench or passing to the wrong bench/+3 s for missing the target area (60×30 cm)/+3 s for ball handling/+2 s to pass the ball from outside the passing zone/+2 s if the ball hits a cone/+1 s for each second taken out of the 43 s allocated to complete the test/—1 s for each pass that hits the 10 cm band in the middle of the target (bonus). Players were asked to complete the test as quickly as possible with the fewest errors to optimize performance in the LSPT, and were permitted two trials, the average of which was used as the performance score [33].

### Stroop Test

The Stroop test has previously been used to assess executive function [34] in youth [35, 36]. The test consists of three tasks and 100 stimuli for each task. The first task *(“Stroop Word")* demanded participants’ to read out words as quickly as possible. The second task affords participants to identify the color (“*Stroop Color*”) and in the third task the participant was asked to write the word of the ink used *(“Stroop Word-Color”)* [35, 36]. Each task lasted 45 s, and the participants were asked to respond verbally. The evaluator asked each participant who made a mistake to correct it before continuing. The score for each task included the number of correct answers.

### Blood Sampling

Venous blood sampling of basal BDNF and IGF-1 was carried out after overnight fasting and were performed 24 h after the last training session between 08.00 and 10.00 am. Ten ml of venous blood was collected in tubes without anticoagulant (for serum determination) and kept at room temperature for 30 min. The serum was then separated by centrifugation at 3000 × *g* for 15 min at room temperature. Serum samples were immediately stored at − 80 °C in Eppendorf tubes. Blood samples were collected in a temperature-controlled room (24° ± 1 °C) [37]. IGF-1 serum concentrations (ng/ml) were determined by GH technique RIA (radioimmunoassay) automatic STRATEC. BDNF serum concentrations (pg/ml) were determined following the manufacturer’s guidelines using the method of enzyme-linked immunosorbent assay (ELISA) (kits from the R&D system. Human-Free BDNF Quantikine Elisa Kit; catalog number: DBD00). Blood sampling occurred during the follicular phase at T1 and T2 for all participants.

### Training Protocols

The intervention period lasted 12 weeks and was conducted over three training cycles, each lasting 4 weeks. The SG group performed ST only during the first 3 weeks of each cycle with two weekly ST sessions and three weekly regular soccer training sessions. No ST was conducted during the fourth week of the training cycle. In week 4, five weekly soccer training sessions were applied. Throughout the intervention period, the active CG performed five weekly soccer sessions and no ST (Fig. 1**)**. Each training session lasted 90 min, with 48 h of rest between sessions and a 15-min warm-up and 5-min cool-down. ST comprised full-body strength exercises (i.e., leg press, hip thrust, squat, bench press, lat pull-down, and calf raises). In addition, throughout the 12 weeks of ST, the intensity increased progressively for appropriate overload. The first ST cycle consisted of 3 sets of 15 repetitions for each exercise with slow movement execution at 40–60% of the 1-RM. The second cycle consisted of 3 sets of 12, 10, 8 repetitions for each exercise at 60–75% of the 1-RM. The third and final cycle consisted of 3 sets of 10, 8, 6 repetitions for each exercise with progressive intensities reaching 85% of the 1-RM. To adjust the training load, 1-RM tests were performed before the start of each training cycle. Exercise technique was standardized across the training sessions. Prior to the intervention, participants received detailed instructions and demonstrations regarding the correct execution of each exercise. The ST [27] exercises were performed with the correct exercise technique, using the prescribed training volume and intensity based on recommendations of professional strength and conditioning specialist. This training program has previously been described by Darragi et al. [27] and Bousselmi et al. [26].

#### Training Load Quantification

A 0–10 OMNI scale was used to rate perceived exertion (RPE) of the participating athletes [38]. The participants provided session RPE (sRPE) used as a measure of internal training load 15 to 20 min after each training session. Training duration per session was used as an external training load marker. Overall training load (TL) was calculated on a daily level by multiplying internal by external training load (i.e., sRPE score multiplied by session duration: TL = sRPE x training session duration [min]).

### Statistical Analyses

All data are presented as means and standard deviations (SD). Data normality was tested and confirmed using the Shapiro–Wilk test. Homogeneity of variances was verified using Levene’s test, and sphericity was assessed using Mauchly’s test. To determine the effects of SG versus CG on selected measures of physical fitness, cognitive function and neuroplasticity, a 2 (groups: SG, CG) × 2 (time: pre, post) analysis of variance (ANOVA) for repeated measures was used. In cases of significant group-by-time interactions, Bonferroni-adjusted and group-specific post hoc tests (i.e., paired sample *t* tests) were calculated. Furthermore, the results of the F test depended on the significance of Mauchly’s sphericity test. If significant, the Greenhouse–Geisser correction was used. Partial eta squared (η_p_^2^) was used as a measure of effect size and interpreted as small 0.01 ≤ η_p_^2^< 0.06, medium 0.06 ≤ η_p_^2^< 0.14, or large η_p_^2^≥ 0.14. Percentage changes from pre- to post-tests were calculated as follows: (post–pre)/pre × 100. The familiarization of the tests was carried out and confirmed by the reproducibility values (test–retest trial) to avoid any practice factor linked to learning. In fact, during the physical test familiarization sessions, the players carried out tests and retests (1-week wash) to check their reliability. To assess test–retest reliability, thresholds for the intraclass correlation coefficient (Cronbach’s ICCs) were computed using two-way mixed model according to the method previously described [39].

Spearman correlation coefficients were computed to plot pre–post deltas for training-induced changes in physical fitness markers (i.e., 1 RM, LSPT) and changes in cognitive function (i.e., Stroop). The level of significance was set at *p* <0.05. The statistical software used was SPSS version 22.

## Results

All players received treatment as allocated. No test or training-related injuries were recorded. Adherence rates amounted to 99.4±1.1% for the SG and 99.0±1.7% for the CG. No statistically significant baseline between-group differences were noted.

The intraclass correlation coefficients (ICC) and coefficients of variation (CV) are presented in Table 2.

**Table 2:** Test–retest reliability expressed as relative intraclass correlation coefficient (ICC) and absolute reliability coefficient of variation (CV) markers

Parameters	ICC	95% CI	%CV
1-RM bench press	0.87	0.53–0.96	14.12%
1-RM lat pull-down	0.95	0.84–0.98	20.11%
1-RM leg press	0.89	0.62–0.97	24.76%
LSPT score	0.64	0.14–0.85	17.49%
Stroop word test	0.74	0.31–0.88	14.79%
Stroop color test	0.79	0.50–0.91	14.82%
Stroop word-color test	0.59	0.02–0.83	24.00%

*1 RM* one-repetition-maximum, *LSPT* Loughborough Soccer Passing Test

### Anthropometrics and Body Composition

Anthropometric and body composition characteristics of the participants are displayed in Table 3. Significant main-time effects were observed for body mass (*p* <0.001; η_p_^2^=0.64), BMI (*p* <0.001; η_p_^2^=0.63), and body fat (*p* =0.002; η_p_^2^=0.39). Significant group-by-time interactions were found for body mass (*p* = 0.039; η_p_^2^= 0.19) and percentage of body fat (% body fat) (*p* = 0.006; η_p_^2^= 0.32). Post hoc tests showed a decrease in body fat in the SG but not the CG (*p* = 0.001; η_p_^2^ = 0.32) and a significant increase in body mass in the SG but not the CG (*p* = 0.001; η_p_^2^ = 0.31).

**Table 3:** Anthropometric and body composition characteristics of the participating young and highly-trained female soccer players (Tier 3)

Variables	Group	T1	T2	% change	ANOVA *p* value (partial η^2^)		
					Main effects		Interaction
					Time	Group	Group x time
Body mass (kg)	SG	55.6±9.3	58.2±9.7	4.7%	<0.001 (0.64)	0.900 (0.00)	0.039 (0.19)
	CG	56.7±7.7	58.0±7.6	2.1%			
BMI (kg.m^−2^)	SG	20.4±3.2	21.4±2.8	4.8%	<0.001 (0.63)	0.771 (0.00)	0.138 (0.10)
	CG	20.9±2.3	21.5±2.3	2.8%			
Body fat (%)	SG	25.6±2.7	24.1±2.9	−5.7%	0.002 (0.39)	0.207 (0.07)	0.006 (0.32)
	CG	26.3±2.4	26.2±1.9	−0.4%			

*BMI* body mass index, *SG* strength training group, *CG* active control group, *T1* Pre intervention, *T2* Post intervention

### Muscle Strength

Results for 1-RM maximal strength tests are displayed in Table 4***. ***Significant group-by-time interactions were observed for 1-RM bench press (*p* = 0.002; η_p_^2^ = 0.38), 1-RM lat pull-downs (*p* <0.001; η_p_^2^ = 0.69), and 1-RM leg press (*p* <0.001; η_p_^2^= 0.47). Post hoc tests revealed significant ST-related increases in 1-RM bench press (*p* <0.001; η_p_^2^=0.41), lat pull-downs (*p* <0.001; η_p_^2^=0.43), and leg press (*p* <0.001; η_p_^2^=0.36) in the SG but not the CG.

**Table 4:** Muscle strength after 12 weeks of strength training in young and highly-trained female soccer players (Tier 3)

Variables	Group	T1	T2	% change	ANOVA *p* value (partial η^2^)		
					Main effects	Interaction	
					Time	Group	Group x time
1-RM bench press	SG	35.7±4.6	44.6±5.8	24.7%	<0.001 (0.64)	0.068 (0.15)	0.002 (0.38)
	CG	35.4±4.8	37.5±4.9	6.5%			
1-RM lat pull-down	SG	22.2±4.0	30.8±5.9	38.5%	<0.001 (0.88)	0.002 (0.38)	<0.001 (0.69)
	CG	18.8±3.9	21.5±3.4	14.0%			
1-RM leg press	SG	64.9±21.0	101.5±27.1	56.5%	<0.001 (0.85)	0.004 (0.34)	<0.001 (0.47)
	CG	50.3±11.5	66.1±11.2	31.5%			

*SG* strength training group, *CG* active control group, 1-RM one-repetition-maximum, *T1* Pre intervention, *T2* Post intervention

### Loughborough Soccer Passing Test

Results for group-specific pre–post LSPT performance are illustrated in Table 5***.*** There were significant main-time effects for the penalty (*p* = 0.001; η_p_^2^=0.45) and score (algebraic sum of LSPT time and LSPT penalty, *p* = 0.004; η_p_^2^=0.34). Significant group-by-time interactions were observed for penalty (*p* = 0.007; η_p_^2^=0.31) and for score (p=0.026; η_p_^2^=0.22). In addition, post hoc tests indicated pre–post decreases in the SG but not the CG for penalty (*p* = 0.003; η_p_^2^=0.36) and score (*p* = 0.007; η_p_^2^=0.22).

**Table 5:** Performance in the Loughborough Soccer Passing Test for young and highly-trained female soccer players (Tier 3)

Variables	Group	T1	T2	% change	ANOVA *p* value (partial η^2^)		
					Main effects		Interaction
					Time	Group	Group x time
Time (s)	SG	49.8±4.5	49.2±5.9	−1.2%	0.792 (0.00)	0.068 (0.15)	0.855 (0.00)
	CG	53.2±3.3	53.1±7.2	−0.2%			
Penalty(s)	SG	23.4±14.9	8.1±5.7	−65.4%	0.001 (0.45)	0.408 (0.03)	0.007 (0.31)
	CG	20.0±6.6	17.7±10.3	−11.4%			
Score(s)	SG	73.2±18.4	57.3±9.6	−21.7%	0.004 (0.34)	0.202 (0.08)	0.026 (0.22)
	CG	73.2±9.5	70.8±15.7	−3.3%			

*SG* strength training group, *CG* active control group, 1-RM one-repetition-maximum, *T1* Pre intervention, *T2* Post intervention

### Executive Function

The Stroop test was used to assess executive function, and results are presented in Table 6. Significant main-time effects were observed for the Stroop word test (*p* <0.001; η_p_^2^=0.64), the Stroop color test (*p* <0.001; η_p_^2^=0.65), and the Stroop word-color test (*p* <0.001; η_p_^2^=0.77). Significant group-by-time interactions were observed for the Stroop word-color test (*p* = 0.006; η_p_^2^=0.32). The post hoc test revealed significantly improved performance in the Stroop Word-Color test in the SG but not the CG (*p* <0.001; η_p_^2^=0.56).

**Table 6:** Performance for Stroop test outcomes in young and highly-trained female soccer players (Tier 3)

Variables	Group	T1	T2	% change	ANOVA *p* value (partial η^2^)		
					Main effects		Interaction
					Time	Group	Group x time
Word test	SG	92.0±11.7	111.3±13.3	20.9%	<0.001 (0.64)	0.356 (0.04)	0.324 (0.04)
	CG	89.9±12.9	103.6±16.4	15.3%			
Color test	SG	61.8±12.1	75.4±8.2	21.9%	<0.001 (0.65)	0.156 (0.09)	0.155 (0.09)
	CG	58.6±7.6	66.9±11.9	14.1%			
Word-color test	SG	35.5±6.8	52.0±7.8	46.7%	<0.001 (0.77)	0.374 (0.04)	0.006 (0.32)
	CG	37.5±5.3	45.1±8.5	20.4%			

*SG* strength training group, *CG* active control group, *T1* Pre intervention, *T2* Post intervention

### Blood Neurotrophins

Changes in serum concentration of BDNF and IGF-1 in the two experimental groups are presented in Table 7. There were no significant main effects of time, group or group-by-time interactions.

**Table 7:** Serum concentration for markers of neuroplasticity in young and highly-trained female soccer players (Tier 3)

Variables	Group	T1	T2	% change	ANOVA *p* value (partial η^2^)		
					Main effects		Interaction
					Time	Group	Group x time
BDNF (pg/ml)	SG	3022.9 ±636.5	3148.7 ±390.5	4.2%	0.574 (0.01)	0.898 (0.00)	0.730 (0.00)
	CG	3038.8 ±792.9	3068.9 ±748.6	1.0%			
IGF-1 (ng/ml)	SG	315.2 ±141.5	310.8 ±103.0	−1.4%	0.231 (0.07)	0.725 (0.00)	0.368 (0.04)
	CG	342.3 ±66.8	311.7 ±60.8	−8.9%			

*SG* strength training group, *CG* active control group, *IGF-1* insulin-like-growth hormone factor1, *BDNF* brain-derived neurotrophic factor, *T1* Pre intervention, *T2* Post intervention

### Associations Between Training-Related Changes in Physical Fitness and Cognitive Function

There were no statistically significant correlations (*p* >0.05, *r* = − 0.118 to 0.451) between measures of cognitive function (i.e., Stroop word color) and performance metrics (i.e., 1 RM and LSPT).

### Training Load

The two groups had similar TL with 1164.8±49.0 arbitrary units (AU) in the SG and 1158.4±67.8 AU in the CG (*p* = 0.793; η_p_^2^=0.00).

## Discussion

To our knowledge, this is the first study to examine the effects of ST on physical fitness, cognitive function, and selected markers of neuroplasticity in young and highly-trained female soccer players. The main findings of this study were that a 12-week in-season ST program with two weekly sessions induced improvements in soccer skills (e.g., LSPT scores) and cognitive function (e.g., Stroop scores). However, there were no significant changes in the selected markers of neuroplasticity (e.g., BDNF and IGF-1). Nonetheless, the ST program did induce a significant increase in muscle strength (e.g., 1 RM of bench press, lat pull-down and leg press).

### Anthropometric and Body Composition Characteristics

Twelve weeks of ST increased significantly body mass and decreased the percentage of body fat in the SG with a large sized effect. Other studies also reported that ST [25] and neuromuscular training [40] improved body composition in elite female soccer players (Tier 4 training and performance caliber). The ST can lead to changes in the body composition of female elite players, such as increasing muscle mass and morphology (e.g., muscle thickness and muscle fiber cross-sectional area) [25], stimulating muscle hypertrophy, and reducing fat mass [40]. However, Lesinski et al. [24] showed no significant difference in body composition among young elite female soccer players (Tier 4 training and performance caliber) after a ST program lasting for a full soccer season. These divergencies between studies can mainly be explained by the applied ST programs. In fact, it is well-known that the efficacy of ST on body composition depends on the intensity, duration, and type of exercises as well as the fitness level of the players at baseline [25].

### Muscle Strength

ST increased maximal strength (1-RM) after 12 weeks, as shown by improved leg press, bench press, and lat pull-downs. Our findings are supported by the study of Lesinski et al. [24], which indicates that power training at moderate to high-intensity levels (50–95% of the 1-RM) significantly increased 1-RM leg press in young female soccer athletes (Tier 4 training and performance caliber). Ruivo et al. [41] reported that 16 weeks of ST increased 1-RM bench press in adolescent male soccer players (Tier 2 training and performance caliber). In their recent systematic review, Zouita et al. [25] demonstrated that ST improved 1-RM performances of different muscle groups in elite female athletes (Tier 4 training and performance caliber). There is evidence that ST improves muscle strength in elite female players, ranging from 8 to 18.9% after 8–12 weeks of training, depending on the initial training status of the players [25]. As the highly-trained female soccer players included in this study practiced ST for the first time, changes in strength were pronounced early during the training program. This may be explained by the fact that ST led to primarily neural adaptations (motor unit recruitment, firing frequency), especially at the beginning of the training (up until weeks 4–6) [22, 25].

### Loughborough Soccer Passing Test

In the current study, 12 weeks of ST improved LSPT scores in the SG, with a percentage change of − 21.7%. The improvement in LSPT scores was due to the decrease in penalties. The decrease in penalties for SG was − 65.36%, compared to − 11.4% for CG. This may be due to improvements in information processing, leading to faster decision-making due to an enhanced cognitive-motor connection, resulting in improved cognitive function. The LSPT test assesses soccer players’ perceptual–cognitive performance [42]. During the test, players must repeatedly make rapid decisions while simultaneously executing accurate ball dribbling skills and controlling their body movements within a confined space [42, 43]. This finding can likely be explained by training-induced improvements in cognitive function, particularly through enhancements in executive functions and cognitive flexibility, as suggested by previous studies [35, 44]. Such mechanisms may contribute to improved learning and memory within the central nervous system [45, 46]. Wen et al. [47] concluded in a literature review that all types of training, including strength and endurance training [48] and training that combines training and agility techniques [49], improved LSPT scores. Likewise, authors of a meta-analysis also reported that ST improves these kinds of performances in female soccer players (Tier 2 training and performance caliber) [50]. The LSPT test also provides information on overall motor performance (LSPT time) as well as fine motor skill levels (LSPT penalty) [42]. Furthermore, in highly-trained young soccer players with Tier 3 training and performance caliber, Ben Ounis et al., [51] observed that total performance during LSPT correlated positively and significantly with 5-m, 20-m, and 30-m linear sprint times, and with different agility test performances (15-m agility test with and without the ball, Illinois agility test (*r* = 49–0.75; *p*<0.01, respectively). As such, the training-induced improvement in selected measures of physical fitness (i.e., 1-RM bench press, lat pull-down, and leg press) of our soccer players may have contributed to enhanced LSPT scores in the form of transfer effects.

### Stroop Test

ST produced significant effects on cognitive function in this study, as shown by the results for the Stroop test. Although the observed gains in the Stroop word-color test were significant they should be interpreted with caution, as part of the observed improvements may be attributable to measurement variability and/or learning effects. The findings of this study on the benefits of ST on Stroop scores are consistent with a meta-analysis conducted by Landrigan et al. [52]. These authors reported improvements in cognitive function following ST. The enhanced cognitive flexibility and inhibition in SG potentially reflect an improved capacity to suppress automatic responses, such as reading the word instead of naming the color of the ink, resulting in shorter reaction times and fewer errors. This may indicate improvements in executive functions that are essential for decision-making and performance under pressure in team sports [53].

According to Park and Etnier [35], exercise has acute effects on Stroop test performance, as moderate-intensity exercise significantly improved information processing in adolescents (Tier 1 training and performance caliber). In addition, high-intensity interval training was more effective than moderate-intensity continuous exercise in improving cognitive function using the Stroop test [28]. It was also demonstrated that improvements in physical performance result from improved cognitive function in young soccer players (Tier 2–3 training and performance caliber) [7]. Several studies reported that playing soccer can enhance neurocognitive abilities, as shown by improvements in the Stroop test [6, 54]. In addition, the cognitive function of adolescent soccer players is also influenced by their fitness levels, so high-fit adolescent soccer players exhibit greater improvements in the Stroop test compared to low-fit adolescent soccer players [6].

Moreover, ST can induce permanent changes in muscle strength and brain plasticity [22]. ST allows for increased excitability and greater recruitment of motor units, which may allow an increase in synaptic efficiency and enhanced corticospinal excitability and corticospinal responses [22]. The resulting changes in neural adaptations can lead to improved cerebral plasticity and cognitive function [22].

### Brain-Derived Neurotrophic Factor and Insulin-Like Growth Factor 1 Basal Concentrations

It is well-known that BDNF increases immediately after an acute exercise bout, regardless of the type of exercise, i.e., aerobic, anaerobic, strength, or high-intensity interval exercise [54–56]. Less is, however, known about the long-term effects of training in general and ST in particular on BDNF secretion. Of note, BDNF is a biomarker of cognitive function and brain health [45, 55, 56] and elevated BDNF secretion levels are associated with improved cognitive function [45]. More specifically, BDNF plays a critical role in neuronal development, survival, and growth in human beings, while there is also preliminary evidence that it may promote neurogenesis within the human hippocampus [45, 46, 57]. Together, these mechanisms support synaptic plasticity and are associated with enhanced learning, memory, and cognitive function in the central nervous system [45, 46, 57]. However, the long-term effects of ST on BDNF concentrations are inconclusive. This study indicates no significant group-by-time interactions. However, there are limited studies on ST effects on basal BDNF concentration. A study by Schiffer et al. [9] found no significant differences in basal BDNF concentration at rest after a 12-week ST program in sports science students (Tier 0 training and performance caliber). Our findings are in agreement with numerous studies that concluded that ST did not change resting concentrations of BDNF [9, 58, 59]. There is no evidence supporting a long-lasting effect of BDNF secretion after ST. Training has an immediate or acute effect on BDNF concentrations and it up-regulates its cellular processing, including synthesis, release, absorption, and degradation [60]. It can be hypothesized that the effects of long-term training on BDNF concentrations are transient [58].

To date, studies on the effects of ST on basal BDNF concentration are inconclusive, as a recent review found no consensus on the positive effects of ST on BDNF concentration in young, healthy adults (Tier 0 training and performance caliber) [61]. There is also uncertainty about the effects of different types of training on BDNF concentration, as shown in the differences in findings in the literature [59, 61]. The secretion of BDNF is regulated by the duration of exercise, the intensity and type of exercise, the frequency of the intervention, and the sex of the study subjects [62], making it difficult to determine optimal exercise conditions to improve BDNF concentration [63]. In addition, BDNF concentrations correlated with BMI, as there are no changes in BDNF concentration after exercise in healthy subjects with a BMI less than 25 kg/m^2^ [59]. The stabilization of BDNF values in this study may be related to an average BMI of less than 25 kg/m^2^ (21.4±2.9 at T2) in our participants.

Authors of numerous studies have reported that individuals with elevated levels of physical fitness and regular participation in exercise show no changes in basal BDNF concentration [6, 54]. This lack of effect may relate to the fact that physical activity does not result in unlimited BDNF secretion, but that high BDNF values stagnate in subjects with regular and elevated level of physical activity, leading to a type of homeostasis [45, 64].

Improvements in cognitive function after exercise training can potentially be explained by several mechanisms, such as changes in some neurochemical and neuroendocrine responses [21]. For example, ST increases the expression of IGF-1 [21] and several mechanisms are stimulated by the IGF-1 such as neuroplasticity in the human brain (e.g., synaptic processes, angiogenesis, synaptogenesis) [21]. Furthermore, IGF-1 secretion increases brain volumes by changes in structural grey matter [21]. In addition, IGF-1 enhances brain function (e.g., increase in reaction time and accuracy for executive function, enhancement in processing capacity, and superior global cognition) [21].

However, in the current study, no significant group-by-time interactions were found for IGF-1. Despite IGF-1 increases after ST, the effects of ST on IGF-1 and the mechanism of improving cognitive performance and brain health are uncertain [45]. However, athletes with regular physical activity had higher concentrations of IGF-1 than inactive subjects [45]. Exercises regulate IGF-1 concentration and maintain it at an optimal level [45] which may explain the fact that we did not find any significant difference before and after resistance training and that our players have reached the optimal level of IGF-1. Differences in training type, intensity, and duration lead to variability in training responses [59] which may also explain our results and the fact that our training protocol differs from those of studies that have found increased concentrations. Further research is required for more information.

## Limitations

There are some limitations in this study: (i) Cognitive flexibility was assessed using a limited set of measures. Additional assessments, such as self-report questionnaires evaluating cognitive and behavioral flexibility in everyday life (e.g., self-regulatory behaviors, adaptability to changing situations, and perceived flexibility), may provide a more comprehensive evaluation of this variable. (ii) Only selected neurotrophic factors were measured. The inclusion of additional blood neurotrophins, such as neurotrophin-3 (NT-3) and neurotrophin-4 (NT-4), could have provided a more complete understanding of the neurobiological mechanisms underlying the effects of ST on cognitive function. (iii) The absence of controls on selected factors (e.g., sleep, stress, fatigue), which may have had an impact on some of our measurements. (iv) We studied only young female soccer players and only one training mode (e.g., ST) without considering other specific cognitive training interventions. (v) The a priori power analysis was conducted based on a Stroop test result [28] despite the fact that our current study includes multiple outcome domains (measurements). In fact, there is a lack of studies conducted with young elite female soccer players (Tier 3 training and performance caliber) on the effects of ST on BDNF secretion or IGF-1. (vi) Although Bonferroni adjustments were applied for post hoc comparisons, the study included multiple outcomes testing across physical, cognitive, and biochemical domains. Consequently, an increased risk of type I error cannot be completely excluded and should be considered when interpreting results. (vii) Differences in pubertal development and menstrual hormonal fluctuations may have contributed to variability in some of the measured outcomes among our participants.

## Conclusions

This study demonstrated that 12 weeks of in-season ST improved muscle strength, soccer skills, and cognitive function in young and highly-trained female soccer players (Tier 3 training and performance caliber). Thus, coaches may incorporate ST during soccer training to increase physical fitness levels and cognitive function in female soccer players. However, more research is needed to confirm our findings. Moreover, the physiological mechanisms underlying these training effects need to be explored in greater detail, such as measuring neurovascular or neuroendocrine responses, with the goal of determining the effects of ST (and other exercise modalities) on cognitive function.

## Supplementary Information

Supplementary Material 1.

## Data Availability

The datasets generated and analyzed during the current study are available from the corresponding author upon reasonable request.

## Abbreviations

SG: Strength training group
CG: Active control group
1-RM: One-repetition maximum
COD: Change-of-direction
BDNF: Brain-derived neurotrophic factor
IGF-1: Insulin-like growth factor 1
LSPT: Loughborough Soccer Passing Test
T1: Pre-tests
T2: Post-tests
BMI: Body mass index
ACSM: American College of Sports Medicine
TL: Training load
CV: Coefficients of variation
CI: Confidence interval
AU: Arbitrary units
ANOVA: Analysis of variance
ICC: Intraclass correlation coefficient
RPE: Rating of perceived exertion
SD: Standard deviations
sRPE: Session-rating of perceived exertion
ST: Strength training
