# Supplementary material for: Effects of Strength Training on Cognitive Function, Brain-Derived Neurotrophic Factor and Insulin-Like Growth Factor 1 in Highly-Trained Young Female Soccer Players
Source: Sports Med Open. 2026 Sep 18;12:138. doi: 10.1186/s40798-026-01103-z (PMC13589019; doi:10.1186/s40798-026-01103-z)
Supplement: Supplementary file 1 [file 40798_2026_1103_MOESM1_ESM.docx]

**Declaration: The articles included in this work originated from the same overarching research study, led by Bousselmi M. and Darragi M., with different publications reporting distinct research questions and outcomes.** For transparency, it should be noted that data presented in this manuscript (Bousselmi et al., under revisionin Sports Medicine – Open) is part of a larger research project. Across the four articles from this research project, the same study population and a similar strength training intervention were used, while each article addressed a distinct research objective. The current study of Bousselmi et al. (under revision in Sports Medicine – Open) focusses on the effects of 12 weeks of strength training on soccer-specific skills, cognitive function, and selected markers of blood neurotrophins (BDNF, IGF-1 concentrations) in young female and highly-trained soccer playerswith Tier 3 training and performance calibre. A previously published article in the journal Sports (Bousselmi et al., 2026) reported the effects of the same intervention on muscle damage biomarkers (LDH, CPK) and inflammatory biomarkers (TNF-α, IL-6). Another article published previously in Sports Medicine – Open (Darragi et al., 2024) examined the effects of the same strength training interventionon soccer-related physical fitness variables and the occurrence/recurrence of injuries in the same population of young and highly-trained female soccer players. A fourth manuscript by Darragi et al. (under revision in BMC Sports Science, Medicine and Rehabilitation) reported soccer-related measures of physical fitness and hormonal profiles following the above-mentioned strength training program in the same population. In summary, these manuscripts (Darragi et al., 2024;Bousselmi et al., 2026; Bousselmi et al., under revision in Sports Medicine – Open; Darragi et al. (under revision in BMC Sports Science, Medicine and Rehabilitation)do not share the same study aims.

**The data set used for the fourarticles is also attached as supplementary materials.**

The four research articles presented in the table below originated from the same randomized controlled trial. Although they were based on the same cohort and intervention, each article addressed a different primary research question and reported distinct outcomes. Participants were randomly allocated to either a strength training group (STG) or an active control group (CG). The intervention was implemented over 12 weeks during the competitive soccer season.The training protocol was identical across all four articles. Specifically, players assigned to the STG performed two additional strength training sessions per week (90 min per session) in addition to their regular soccer training schedule, which consisted of three weekly sessions. A minimum recovery period of 48 hours was maintained between strength training sessions. In contrast, participants in the active CG continued their usual soccer training program, comprising five regular soccer training sessions per week. As a result, the overall training volume was comparable between the two groups.

| **Authors; Title; Journal; Year of publication.** | **Objectives** | **Difference in sample sizes across the fourarticles**  **(more details in the excel file)** |
| --- | --- | --- |
| Darragi M, Zouhal H, Bousselmi M, Karamti HM, Clark CCT, Laher I, Hackney AC, Granacher U, Zouita, AB. Effects of in-season strength training on physical fitness and injury prevention in North African elite young female soccer players. Sports Med Open. 2024; 10(1):94. <https://doi:10.1186/s40798-024-00762-0>.  <https://link.springer.com/article/10.1186/s40798-024-00762-0#citeas> | Effects of a 12-week in-season strength training on soccer- related measures ofphysical fitness and the occurrence/recurrence of injuriesin young elite female soccer players. | A total of 30 players were initially enrolled in the study. During the intervention period, four players were excluded:  - 3 players were excluded out of the active CG because of an injury.  - 1 player was excluded from the STG because of an injury.  The final sample consisted of 26 participants, with 13 players allocated to each group. |
| Bousselmi M, Zouita ABM, Darragi M, Karamti HM, Zouita S, Del Coso J, Hmid AB, Hackney AC, Granacher U, Zouhal H. Twelve weeks of in-season strength training at moderate intensities improve strength and body composition without increasing muscle damage or inflammation in elite young female soccer players. Sports (Basel).2026; 14(4):136.<https://doi:10.3390/sports14040136>.  <https://www.mdpi.com/2075-4663/14/4/136> | Effects of a 12-week in-season strength training program on maximal dynamic strength, markers of muscle damage (LDH, CPK), and inflammation (TNF-α, IL-6) in young elite female soccer players. | Thirty players were initially enrolled in the study.  Four players were excluded from the active CG:  - 1 players' blood analyses could not be analyzed because the cryotubes were destroyed during serum thawing for the TNF-α tests,  - 3 player had an injury.  Two players were excluded from the STG:  - 1 player because there was no blood sample available for analysis,  - 1 player because of an injury.  As a result, 24 players (12 participants in each group) made up the final sample. |
| BousselmiM,Zouhal H, Darragi M, Karamti HM, Ben Hmid A, Zamali I, Ben Ahmed M, Krir A, Zouita S, Laher I, Hackney AC, Granacher U, Zouita A. Effects of strength training on cognitive function, brain-derived neurotrophic factor and insulin-like growth factor 1in highly-trained young female soccer players. Under review in Sports Med- Open. 2026. | Effects of 12 weeks of strength training on physical fitness, soccer-specific skills, and cognitive function, and selected markers of blood neurotrophins(BDNF, IGF-1 concentrations) in young female soccer players. | Thirty players were initially enrolled in the study.  Five players were excluded from the active CG:  - 2 players' blood analyses could not be analyzed because the cryotubes were destroyed during serum thawing for the TNF-α tests,    - 3 player had an injury.  Three players were excluded from the STG:  - 2 players because there was no blood sample available for analysis,  - 1 player because of an injury.  As a result, the analysis was limited to 22 participants. |
| Darragi M**,** Zouita AB, Bousselmi M, Kerir A, Karamti HM, Saeidi A, Hackney AC, Granacher U, Zouhal H. Physical fitness and hormonal responses to strength training in adolescent elite female soccer players.Under review in BMC Sports Sci Med Rehabil. 2026. | Effect of a 12-week in-season strength training program on soccer-related measures of physical fitness and hormonal profiles in young elite female soccer players. | Thirty players initially participated in the study.  Four players were excluded from the active CG:  - 1 player due to incomplete blood measurements  - 3 player had an injury.  Two players were excluded out of the STG:  - 1 player because incomplete blood measurements  - 1 player had an injury.  As a consequence, 24 players total, 12 in each group, made up the final analytic sample. |

CG: active control group, STG: strength training group, LDH: Lactate dehydrogenase, CPK: Creatine phosphokinase, IL-6: interleukin 6, TNF-α: Tumor necrosis factor alpha, IGF-1: insulin-like growth factor 1 BDNF: Brain-Derived Neurotrophic Factor

**References**

**Darragi M,** Zouhal H, Bousselmi M, Karamti HM, Clark CCT, Laher I, Hackney AC, Granacher U, Zouita, AB. Effects of in-season strength training on physical fitness and injury prevention in North African elite young female soccer players. Sports Med Open. 2024;10(1):94. <https://doi.org/10.1186/s40798-024-00762-0>

**Bousselmi M,** Zouita ABM, Darragi M, Karamti HM, Zouita S, Del Coso J, Hmid AB, Hackney AC, Granacher U, Zouhal H. Twelve weeks of in-season strength training at moderate intensities improve strength and body composition without increasing muscle damage or inflammation in elite young female soccer players. Sports (Basel). 2026;14(4):136. <https://doi.org/10.3390/sports14040136>

**Bousselmi M,**Zouhal H, Darragi M, Karamti HM, Ben Hmid A, Zamali I, Ben Ahmed M, Krir A, Zouita S, Laher I, Hackney AC, Granacher U, Zouita A. Effects of strength training on cognitive function, brain-derived neurotrophic factor and insulin-like growth factor 1in highly-trained young female soccer players.Under revision in Sports Med Open.2026.

**Darragi M,** Zouita AB, Bousselmi M, Kerir A, Karamti HM, Saeidi A, Hackney AC, Granacher U, Zouhal H. Physical fitness and hormonal responses to strength training in adolescent elite female soccer players.Under revision in BMC Sports Science, Medicine and Rehabilitation. 2026.
